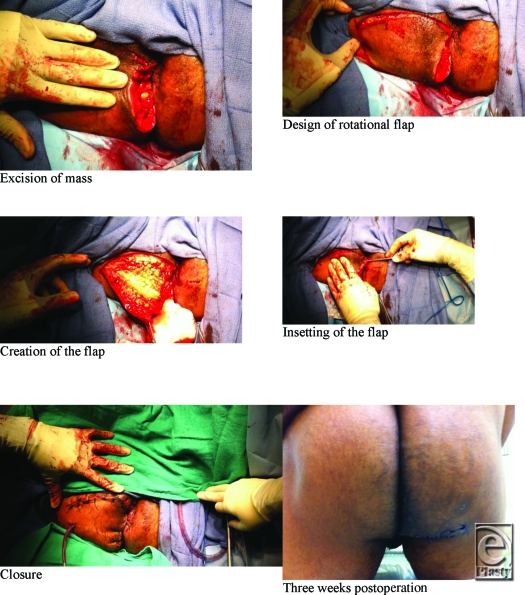# Perianal Mass

**Published:** 2011-04-11

**Authors:** Neilendu Kundu, William Abouhassan, Christopher Ibikunle

**Affiliations:** ^a^Departments of General Surgery; ^b^Plastic Surgery, Cleveland Clinic, Cleveland, OH

## DESCRIPTION

28M presented to the surgery clinic with a several week history of difficulty deficating and bright red blood per rectum. He had significant rectal pain, which limited his examination. Thus, the decision was made to go to the operating room for an examination under anesthesia. Upon evaluation, the above images were appreciated.

## QUESTIONS

1. **What is the differential diagnosis of a perianal mass?**
2. **What is the lymphatic drainage of the rectum?**
3. **What are the risk factors of squamous cell carcinoma of the anus?**
4. **What is the appropriate workup/treatment of perianal squamous cell carcinoma?**
5. **What are the types of closure useful for these types of lesions?**

## DISCUSSION

Perianal masses are fairly rare, accounting for approximately 2% of all colorectal cancers. Most common presentations occur in men. Squamous cell carcinoma (SCC) of the anus typically presents as a bleeding mass with pain or tenesmus. These lesions are large and typically have central ulceration, which can be present for several years prior to presentation. A form of perianal SCC known as Bowen's disease (high-grade intraepithelial SCC) is often associated with condylomas. Less than 10% of patients with Bowen's disease develop invasive squamous cell carcinoma of the anus. Basal cell carcinoma presents in a similar fashion to other perianal carcinoma. Paget's disease (intraepithelial adenocarcinoma) is mainly seen in women in the seventh or eighth decade of life who present with intractable anal pruritis. Biopsy confirmation is utilized and a thorough workup for occult malignancy is required as a large proportion have a coexistent gastrointestional carcinoma.

Lymphatic drainage of the anal canal is divided depending on the location to the dentate line. Lymphatics affecting the anal margin (distal to the dentate line) drain to the inguinal nodes, although it can also drain to the superior or inferior rectal nodes. Lymphatics affecting the anal canal (proximal to the dentate line) drain cephalad to the inferior mesenteric nodes via the superior rectal lymphatics. They also drain laterally and inferiorly to the internal iliac nodes through the ischiorectal fossa via middle and inferior rectal vessels.

The highest rates of anal SCC were historically found in women until the past decade. However, in the past decade, men younger than 45 who have sex with men (anal receptive) have the greatest incidence and number of reported cases. Some risk factors for SCC of the anus include human papillomavirus infections (HPV), smoking, and immunocompromised states (eg, human immunodeficiency virus [HIV]). While precursor lesions (anal intraepithelial neoplasia) exist, it is not thought to be a risk factor. HPV is linked directly via mucoepithelial histological alterations. Smoking is a defined risk factor independent of sexual practices. HIV does not have an understood pathway for its role as a risk factor in SCC; however, it has been noted in several epidemiological studies.

Patients with anal SCC most often undergo neoadjuvant therapy prior to surgical excision. According to DeNardi, sentinel lymph node biopsy has shown to be a more accurate method for staging although the presence of regional nodal metastases is a poor prognostic indicator. Staging is further completed by computer tomographic images of the chest, abdomen, pelvis, and transanal ultrasound for assessment of the depth of invasion. The standard of care for SCC of the anus is chemoradiation with 5-FU and cisplatin or mitomycin-C. Small (<1 cm) well-differentiated cancers without evidence of lymphovascular invasion may be excised primarily if negative margins are achieved without sphincter compromise.

The perineum utilizes the Gluetus Maximus myocutaneous flap as the predominant workhorse in this anatomic area. While Gracilis flaps provided success in some trials, the gluteus maximus flap has been repeatedly demonstrated to be superior. Oftentimes, a significant amount of tissue is not required and simple advancement flaps can be fashioned without detrimental cosmesis. Gluteus maximus fasciocutaneous or myocutaneous flaps can be utilized to reconstruct the sphincter.

## Figures and Tables

**Figure:**
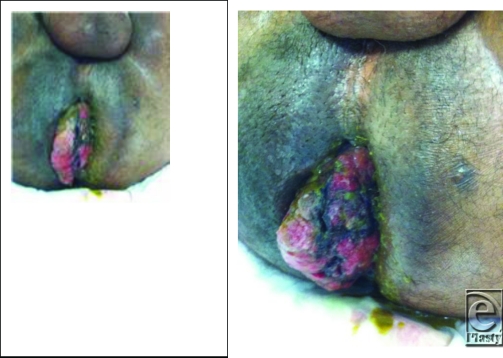


**Figure:**